# Tissue-Wide Gene Expression Analysis of Sodium/Phosphate Co-Transporters in Pigs

**DOI:** 10.3390/ijms20225576

**Published:** 2019-11-08

**Authors:** Aisanjiang Wubuli, Henry Reyer, Eduard Muráni, Siriluck Ponsuksili, Petra Wolf, Michael Oster, Klaus Wimmers

**Affiliations:** 1 Leibniz Institute for Farm Animal Biology (FBN), Wilhelm-Stahl-Allee 2, 18196 Dummerstorf, Germany; wubuli@fbn-dummerstorf.de (A.W.); reyer@fbn-dummerstorf.de (H.R.); murani@fbn-dummerstorf.de (E.M.); ponsuksili@fbn-dummerstorf.de (S.P.); oster@fbn-dummerstorf.de (M.O.); 2 Nutrition Physiology and Animal Nutrition, University of Rostock, Justus-von-Liebig-Weg 6b, 18059 Rostock, Germany; petra.wolf@uni-rostock.de; 3 Animal Breeding and Genetics, University of Rostock, Justus-von-Liebig-Weg 7, 18059 Rostock, Germany

**Keywords:** phosphorus homeostasis, pig, tissue-specific transcript rate, transcellular Na+/Pi co-transporter

## Abstract

Sodium/phosphate co-transporters are considered to be important mediators of phosphorus (P) homeostasis. The expression of specific sodium/phosphate co-transporters is routinely used as an immediate response to dietary interventions in different species. However, a general understanding of their tissue-specificity is required to elucidate their particular contribution to P homeostasis. In this study, the tissue-wide gene expression status of all currently annotated sodium/phosphate co-transporters were investigated in two pig trials focusing on a standard commercial diet (trial 1) or divergent P-containing diets (trial 2). A wide range of tissues including the gastrointestinal tract (stomach, duodenum, jejunum, ileum, caecum, and colon), kidney, liver, bone, muscle, lung, and aorta were analyzed. Both trials showed consistent patterns in the overall tissue-specific expression of P transporters. While SLC34A2 was considered as the most important intestinal P transporter in other species including humans, SLC34A3 appeared to be the most prominent intestinal P transporter in pigs. In addition, the P transporters of the SLC17 family showed basal expression in the pig intestine and might have a contribution to P homeostasis. The expression patterns observed in the distal colon provide evidence that the large intestine may also be relevant for intestinal P absorption. A low dietary P supply induced higher expressions of *SLC20A1*, *SLC20A2*, *SLC34A1*, and *SLC34A3* in the kidney cortex. The results suggest that the expression of genes encoding transcellular P transporters is tissue-specific and responsive to dietary P supply, while underlying regulatory mechanisms require further analyses.

## 1. Introduction

Phosphorus (P) is essential for all living beings. It plays an important role in bone formation, energy metabolism (i.e., ATP), cell signaling (i.e., phosphorylation), cell membranes, blood buffering, and any processes involving nucleic acids [1]. In fact, monogastric farm animals need sufficient nutritional P supply for growth processes and to develop a healthy skeletal system. However, an inefficient and excessive supplementation of mineral P interferes with the natural P-cycle and might cause high environmental P loads [2,3]. Considering the importance of pig for the global meat production, strategies to balance environmental issues and animal welfare have to be identified. Therefore, an improvement in global P efficiency including farm animal husbandry is desirable to achieve a sustainable P-cycle. A deeper understanding of animal-intrinsic P flows can contribute to this matter.

The utilization of P in livestock species is influenced by several factors, one of which is genetics [4,5]. Specifically, heritability estimates indicate that 42% of phenotypic variations in hematological P values of pigs are explained by genetics [6]. In particular, underlying genes might affect the gastrointestinal absorption of P, processes within the organism and cells, storage and usage of P in skeletal tissues, and the retention of P in the kidney [7,8]. Accordingly, promising candidate genes, which are frequently the subject of analyses in studies focusing on the regulation and maintenance of the P homeostasis, are sodium/P co-transporters [9,10].

Phosphate absorption is mediated by passive paracellular and active transcellular mechanisms. The latter serve e.g., for the directed enteral absorption and renal excretion of P, which is achieved via P transporting proteins. Various isoforms of these P transporters are known to be tissue-specifically expressed. In intestinal tissues, P transporters are located on the apical luminal sides of epithelial cells. In the kidney, they occur mainly in the proximal tubules. It has been shown that their gene expression is controlled by hormones such as parathyroid hormone (PTH), fibroblast growth factor-23 (FGF23), and calcitriol, which ensure transcellular P transport depending on dietary supply [11,12].

So far nine genes from three independent subfamilies, including *SLC17A1, SLC17A2, SLC17A3, SLC17A4, SLC20A1, SLC20A2, SLC34A1, SLC34A2*, and *SLC34A3* were annotated as sodium-dependent P co-transporters in pigs (Table 1). The members of the SLC17 family (also called type I sodium/phosphate co-transporter) were initially identified as sodium-dependent P co-transporters. However, recent evidence showed that their P transport activity is relatively weak compared to the other P transporter families [13]. Co-transporter of the SLC34 family (also called type II sodium/phosphate co-transporter) are considered to be major contributors to P homeostasis and are known to be expressed in a wide set of tissues [14]. The co-transporter of the SLC20 family (also called type III sodium/phosphate co-transporter) was initially identified as retroviral receptors, but later studies showed that they act as sodium-dependent P co-transporter [15]. All these P transporters were found in primate and rodent species, including humans and mice with relatively high sequence homology. However, according to current genome database only a part of them were found in *Sauropsida* (reptile and bird species). Although great efforts have been made to uncover molecular mechanisms of P homeostasis, a comparative tissue-wide gene expression profile of these transporters is still lacking in pigs. This will lead to a better understanding of how these P transporters contribute to P homeostasis and respond to different levels of nutritional P feeding conditions in different tissues.

This study was designed to provide new insights into the tissue-specific gene expression of sodium/phosphate co-transporters in pigs. The analysis focuses on the transcriptional level as the primary control site for the expression of genes and as the basis for the repertoire and abundance of the corresponding gene products. The regulatory role of the transporters in P homeostasis is addressed in pigs with different dietary P supplies. Accordingly, two independent pig experiments were analyzed. The first trial focuses on a standard commercial diet (Trial 1), whereas in the second trial the responses to low and high P diets (Trial 2) were investigated.

## 2. Results and Discussion

**Figure 1:**
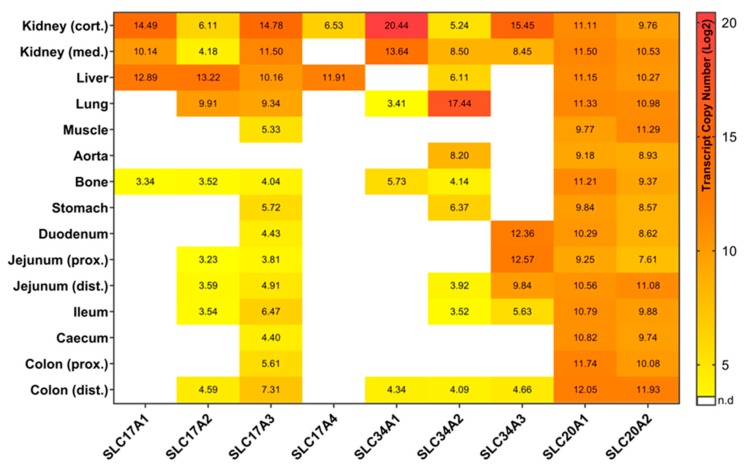
Heatmap of the gene expression of nine sodium/phosphate co-transporters in pigs on a standard commercial diet (Trial 1). In total five German Landrace pigs were fed a standard P diet for six months. Transcript copy numbers of all nine sodium/phosphate co-transporter were measured in 15 tissues by qRT-PCR. Copy numbers were displayed as log2 values.

**Figure 2:**
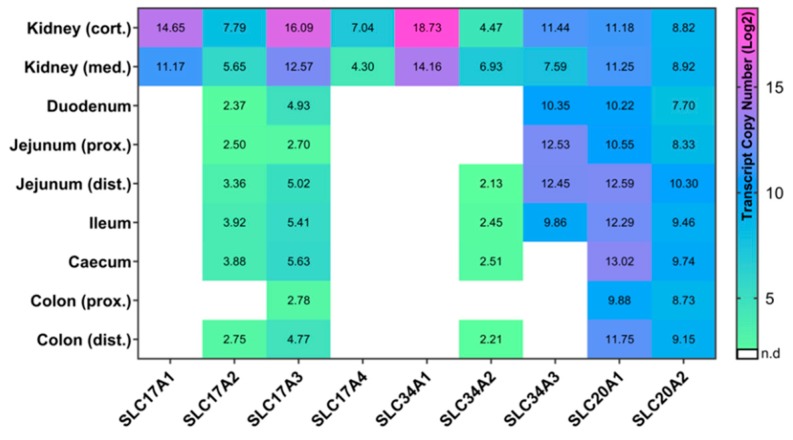
Heatmap of the integrated gene expression levels of nine sodium/phosphate co-transporters in pigs receiving divergent P-containing diets (Trial 2). In total 10 growing pigs were fed a high and low P diet for four month. Transcript copy numbers of all nine sodium/phosphate co-transporter were measured by qRT-PCR. In this heatmap, the average number of copies was calculated across all 10 pigs. Copy numbers were displayed as log2 values.

**Figure 3:**
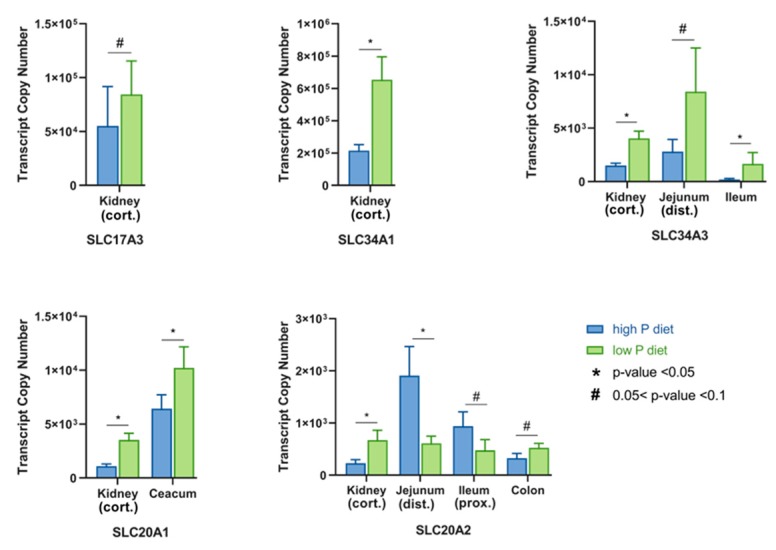
Transcript copy numbers of differentially expressed P transporters in pigs receiving divergent P-containing diets (Trial 2). Sodium/phosphate co-transporters differentially expressed between animals fed high and low P diets were identified. Superscripts indicate statistically significance (*, *p* < 0.05) and trend (#, *p* < 0.1).

This study comprised of two datasets in order to provide an overview of the tissue-specific abundance of all sodium/phosphate co-transporters currently annotated in pigs. Trial 1 serves as an explorative data set to elucidate the absolute expression of P transporters in 15 different tissues and tissue compartments under basal conditions. While almost all transporters were abundant in kidney cortex, kidney medulla, and liver, specific expression patterns occur in tissues of the gastrointestinal tract and peripheral tissues (Figure 1). Trial 2 was designed to examine the effects of different levels of dietary P on the mRNA expression of these P transporter genes in P absorbing and reabsorbing tissues of growing pigs to distinguish dietary responses from constitutive abundance. In addition, all data from trial 2 were integrated to get insights into putative age- and breed-related differences in the P transporter expression profiles (Figure 2). In general, the expression profiles of the two trials were basically consistent. In addition, some of the sodium/phosphate co-transporters showed different expression patterns between high and low P group (Figure 3).

### 2.1. Type I Sodium/Phosphate Co-Transporters Expression Analysis

Type I sodium-dependent P co-transporters are represented by four members: *SLC17A1*, *SLC17A2*, *SLC17A3*, and *SLC17A4*. In trial 1, *SLC17A1* has shown a limited expression profile in the 15 tissues, with the highest expression level in the kidney cortex and the lowest detectable expression in the bone (Figure 1). In trial 2, *SLC17A1* expression in the kidney was confirmed. For both trials no expression was measurable in the gastrointestinal tract. Previous studies revealed similar results, but also showed *SLC17A1* expression in brain samples, which were not included in the current study [16,17,18]. The expression of *SLC17A1* in bone tissue has not yet been described in other studies. The current results indicate that SLC17A1 might be responsible for the transport of uric acid from the liver to the serum and kidney. Indeed, *SLC17A1* expression was linked to renal urate export [19], and the transport of P across hepatic basolateral membranes [20,21]. Although SLC17A1 was initially identified as a sodium-dependent P co-transporter, this role is questioned due to the low level of its P binding affinity [22]. Current investigations point to other functions such as an organic anion transporter [23] or as a chloride-dependent P modulator [24,25]. However, in a knock-out mice study, changes in *SLC17A1* expression still seem to affect renal P homeostasis [26], requiring the clarification of the detailed mechanisms.

*SLC17A2* showed a wide expression profile in both the peripheral and intestinal tissues in the two trials (Figure 1 and Figure 2). Interestingly, its expression is considerably lower in the kidney compared to *SLC17A1* but showed relatively high expression levels in liver and lung tissue. In fact, previous studies also reported that the *SLC17A2* gene was expressed in a wide range of tissues including heart, muscle, kidney, liver, and lung [27]. It has multiple transport functions such as chloride-dependent anion transport, sodium-dependent P uptake, and poly-specific organic anion transport involved in urate circulation [27,28]. According to the expression profiles of both trials, SLC17A2 might further contribute to intestinal P absorption, albeit being low abundant in these compartments. Between the two trials some differences in expression profiles were observed, mainly due to lower detection limits.

The third type I sodium/phosphate co-transporter, *SLC17A3*, showed the broadest expression profile among the SCL17 family members in both trials (Figure 1 and Figure 2). It showed relatively high expression values in kidney, liver, and lung and was solely not detectable in aorta. Overlapping findings are found in the Human Protein Atlas [29]. Due to its abundance in different tissues, SLC17A3 might act as a multi-specific organic anion transporter. SLC17A3 is known to be localized in the renal tubular cells and functions as a voltage-driven urate transporter [30]. Moreover, it seems to represent an excretory route for organic anionic agents as well as urate in vivo, which has been detected in hyperuricemia patients with mutated SLC17A3 [31]. Interestingly, in both trials the *SLC17A3* gene is constitutively expressed in all segments of the gastrointestinal tract, which is known to play an important role in P uptake and transport (Figure 1 and Figure 2). In addition, the expression of *SLC17A3* tended to be different in the kidney cortex of pigs fed divergent P-containing diets (Figure 3). Pigs receiving lower dietary P showed higher levels of renal (cortex) *SLC17A3* expression compared to the high P group.

The last member of Type I sodium/phosphate co-transporters, *SLC17A4*, showed a very limited expression in both trials. It was only expressed in the kidney and liver (Figure 1 and Figure 2). Interestingly, this differs from findings in humans and rodents, where *SLC17A4* is specifically expressed in the gastrointestinal tract and acts as a polyspecific organic anion exporter with putative involvement in urea extrusion [29,32,33]. However, the results of the current study argue for very specific functions of SLC17A4 exclusively in the liver of pigs, although such a role is not yet described.

### 2.2. Type II Sodium/Phosphate Co-Transporters Expression Analysis

The Type II sodium/phosphate co-transporter family contains three members: *SLC34A1*, *SLC34A2*, and *SLC34A3*. They are considered as the major contributors of P homeostasis in mammals. To date, SLC34A1 and SLC34A3 have been regarded as important players in inorganic P homeostasis in the kidney, while SLC34A2 was suggested to be the most important inorganic P transporter in the intestine [8,34,35].

In the current study, *SLC34A1* was expressed in both trials mainly in the kidney and reached highest expression values among all tested genes (Figure 1 and Figure 2). In mice, SLC34A1 has been shown to be responsible for up to 70% of renal P absorption [7]. Moreover, mutations of the *SLC34A1* gene are known to impaired P homeostasis in mice [9]. Interestingly, beside some abundance in lung and bone, *SLC34A1* was also detectable in the distal colon of trial 1, although only at a low level (Figure 1). The expression level of *SLC34A1* in the kidney cortex is different at a statistically significant level between high and low P groups, with more than three times higher values in the low P group compared to high P group (Figure 3). This observation is in accordance with a previous study focusing on divergent P supply in pigs [4]. Moreover, growing pigs on a low dietary P level exhibit lower serum P levels than those receiving a diet with recommended amounts of P [36]. Therefore, by increasing the renal *SLC34A1* mRNA abundance, the organism attempts to reabsorb P to maintain blood P levels.

*SLC34A2* is expressed in both studies in different parts of the gastrointestinal tract and the periphery including the kidney (Figure 1 and Figure 2). The degree of expression of *SLC34A2* in the kidney is considerably lower compared to the other two members of the SLC34 family. However, it is interesting that although SLC34A2 has been analyzed as the main actor of intestinal P absorption in rodents and humans [8,37], its expression levels in the intestinal tissue of pigs are relatively low in both trials. In addition, the *SLC34A2* gene is highly abundant in the lung, which is in accordance with other studies of rat and human [38,39]. Notably, it is abundant in stomach and aorta at relatively high levels.

In the two trials, the expression profiles of *SLC34A3* show specificity for segments of the small intestine as well as for the kidney (Figure 1 and Figure 2). In intestine the expression values were highest in the duodenum and jejunum, decreased slightly in the ileum and were no longer detectable in the caecum and proximal colon, but were detectable again in the distal colon in trial 1. The small intestine, in particular the jejunum and duodenum are considered to be the main sites of P absorption in the intestinal tract [40,41]. Together, it seems that SLC34A3 may contribute for inorganic P absorption in both the renal and intestinal tract of pigs [42]. Similarly, mutations in *SLC34A3* suggested that this gene plays a key role in maintaining P homeostasis in humans [43,44]. In contrast, SLC34A3 in mice is exclusively expressed in the kidney and was not detected in the intestine [35,45], although similar renal expression profiles were found in mouse and pigs [4,46]. In addition, the expression of *SLC34A3* seems to be responsive to dietary P supply. Pigs from the low P group showed considerably higher *SLC34A3* expression in kidney, distal jejunum, and ileum compared to high P group (Figure 3). This is in accordance with an increased renal reabsorption and intestinal uptake of P with the aim of maintaining blood P values in pigs fed a low P diet.

### 2.3. Type III Sodium/Phosphate Co-Transporters Expression Analysis

The type III sodium/phosphate co-transporter family consists of two members: *SLC20A1* and *SLC20A2*. Both *SLC20A1* and *SLC20A2* genes are ubiquitously expressed in both trials of this study in all peripheral and intestinal tissues at relatively high levels (Figure 1 and Figure 2). In fact, they are known as ubiquitously expressed genes in mammalian cells and were therefore regarded as “housekeeping” transporters of inorganic P to the cells [47,48]. It has been reported that type III sodium/phosphate co-transporters have dual functions both as viral receptors and as sodium/phosphate co-transporters [49,50,51]. Moreover, SLC20A1 and SLC20A2 are the major factors for P homeostasis in the brain of mice and humans, and SLC20A2 is also crucial for maintaining adequate P levels in the cerebrospinal fluid [52,53]. Recently, many studies have provided growing evidences that the SLC20A1 has multiple functions beyond its previously reported role as sodium/phosphate co-transporter. It has been shown that various cellular processes such as normal cell division and proliferation, cell density, cell apoptosis, and many other processes that are also independent of its P transport activity require a certain level of SLC20A1 [54,55,56]. Recently, Couasnay and co-workers identified functions of SLC20A1 independent from P transport for endoplasmic reticulum homeostasis, chondrocyte survival, and skeletal development [57]. In addition, Bon and colleagues have recently reported that SLC20A2, but not SLC20A1, is necessary for the corresponding P-dependent secretion of bone-derived fibroblast growth factor 23 (FGF23), which regulates serum P levels [58]. In trial 2 of the current study, *SLC20A1* and *SLC20A2* were differentially expressed in some tissues in the high and low P group (Figure 3). *SLC20A1* showed a higher expression in the kidney cortex and caecum in the low P group compared to the high P group. However, *SLC20A2* transcript rates were increased in the kidney cortex and proximal colon, but decreased in the distal jejunum and ileum when comparing low P and high P groups.

## 3. Materials and Methods 

### 3.1. Animals

Animal trials in this study were approved by the Scientific Committee of the Leibniz Institute for Farm Animal Biology (FBN). The experimental setup was generally licensed and approved by the ethics committee of the federal state of Mecklenburg-Western Pomerania, Germany (Landesamt für Landwirtschaft, Lebensmittelsicherheit und Fischerei). It was registered under the license LALLFM-V/TSD/7221.3–1-053–15 (16 Dec 2015). The tissue sets for expression analysis were generated in two trials. The first part of the study comprised five German Landrace fattening pigs, which were fed ad libitum a conventional diet according to recommendations [59] (Trial 1). The pigs (two females and three castrates) were housed for six month resulting in an average body weight of 118.4 ± 1.7kg. The second part of the study focused on P-divergent diets (Trial 2). In total 10 hybrids form a German Landrace × Large White × Pietrain cross were fed with P divergent diets from weaning (28 days postnatal) until slaughter (four months). The average body weight at slaughtering was 95.3 ± 10.0 kg. Five pigs (three males and two females) were fed with diets containing low mineral P (L) and five piglets (three males and two females) received diets containing high mineral P (H). From weaning to day 70, the achieved P and calcium levels were 5.2 g/kg and 9.8 g/kg (L) and 7.8 g/kg and 9.1 g/kg (H). In the finishing period, levels for P and calcium were 4.1 g/kg and 6.5 g/kg (L) and 7.0 g/kg and 6.7 g/kg (H). Neither phytase nor other phosphatases were added. Pigs had ad libitum access to pelleted feed and water.

### 3.2. Tissue Sampling

For trial 1, pigs were slaughtered at the age of six month in the experimental slaughterhouse of FBN. They were anaesthetized by electrical stunning and subsequently sacrificed by exsanguination. A total of 15 tissues were sampled according to Table 2 focusing on gastrointestinal tissues (stomach, duodenum, jejunum, ileum, caecum, and colon), kidney, liver, bone, muscle, lung, and aorta. Using the same procedure, the 10 pigs of trial 2 were slaughtered at the age of four month. Here nine tissues with focus on P absorption (gastrointestinal tract) and P excretion (kidney) were sampled (Table 2). Gastrointestinal parts were washed with 0.9% NaCl to ensuring removal of residual digesta. Sampling positions are indicated in Table 2. All samples were cut in pieces and frozen in liquid nitrogen immediately. Samples were stored at −80 °C until downstream analysis.

### 3.3. RNA Isolation and cDNA Synthesis

Total RNA was isolated from all tissues by TRI Reagent according to user guides (Sigma-Aldrich, Taufkirchen, Germany) and treated with Baseline-ZERO DNase mix (Biozym, Hessisch Oldendorf, Germany) for ensuring the removal of any genomic DNA residuals. The DNase treated RNA was purified with the column-based NucleoSpin RNA II-Kit (Macherey–Nagel, Düren, Germany). The concentration of final purified RNA was measured by the NanoDrop 2000 photo-spectrometer (PEQLAB, Erlangen, Germany). The existence of genomic DNA contamination was checked by PCR amplification of the ubiquitously expressed porcine ACTB (forward primer: 5’-GAGAAGCTCTGCTACGTCGC-3’; reverse primer: 5’-CCTGATGTCCACGTCGCACT-3’) and subsequent visualization on 2–3% agarose gel. For each sample, first-strand cDNA were synthesized from 1500 ng total RNA using random primers (Promega, Fitchburg, WI, USA) and oligo d(T) primers in the presence of Superscript III reverse transcriptase (Invitrogen, Karlsruhe, Germany). Existence of genomic DNA contamination in cDNA was also checked again by PCR amplification of porcine ACTB as mentioned above.

### 3.4. Quantitative Real-Time PCR (qRT-PCR)

Primers for all P transporter genes were designed using sequence information from the Ensembl database (accessed on February 2018) and the NCBI primer blast online tool (Supplementary Table S1). For each gene of interest primers for two amplicons were designed. Amplicons from a longer fragment were used to generate reference standard curves to allow the absolute quantification of copy numbers. The shorter nested fragment was intended for real-time PCR amplification of the respective P transporters. Performance of primers and an initial assessment of optimal amplification conditions were identified by end-point PCR. Standards were prepared by PCR amplification using SupraTherm Tag polymerase (GeneCraft, Lüdinghausen, Germany) and the following cycling conditions: An initial denaturation step at 95 °C for 3 min followed by 40 cycles consisting of denaturation at 95 °C for 15 s, annealing at corresponding annealing temperature for 60 s and extension at 72 °C for 60 s. PCR products of standards were checked by 2–3% agarose gel and purified using magnetic beads (Beckmann Coulter, Krefeld, Germany). Concentrations of standards were measured using the NanoDrop 2000 photo-spectrometer.

Gene expression levels of all nine sodium/phosphate co-transporters and RPL32 as a housekeeping gene were quantified by qRT-PCR. Transcript copy numbers of every individual sample (two technical replicates per sample) were measured by the LightCycler 480 SYBR Green I Master system (Roche, Mannheim, Germany) according to the user guides. In detail, the reaction mix contained 6 µL of SYBR Green Master I mix, 0.6 µL of each primer, 2.8 µL of nuclease free water, and 2 µL cDNA. PCR were performed on the LightCycler 480 system.

The amplification program was set as follows: an initial denaturation at 95 °C for 5 min followed by 45 cycles consisting of denaturation at 95 °C for 10 s, annealing at the corresponding annealing temperature (Supplementary Table S1) for 15 s, and extension at 72 °C for 25 s. Transcript copy numbers for each sample were calculated based on standard curve method that uses the cycle threshold values of serial dilutions (10^7^–10^0^ copies) of the corresponding standard. Melting curve analysis was used to check amplified products.

### 3.5. Data Analysis

All data were analyzed by the open sourced R software (v.3.2.3; R Foundation for Statistical Computing, Vienna, Austria). Transcript copy numbers were normalized based on the expression of the housekeeping gene RPL32 and transformed by log2. The lower detection limits were adapted to the sample size. Accordingly, a mean copy number below 8 (log2 = 3) for trial 1 and a mean copy number below 4 (log2 = 2) for trial 2 were considered as non-detectable (n.d). For each tissue, transcripts should be detectable in at least 50 percent of the samples in order to be considered for subsequent data analysis. Tissue-specific numbers of transcript copies were averaged and represented as heatmap using GraphPad Prism 8 (GraphPad Software, San Diego, CA, USA). For trial 2, the comparison of groups on a low and high P diet was performed using a linear regression model (R package stats v3.6) including dietary group as fixed effect and time of slaughter as covariate. Differences were considered as statistically significant at *p* < 0.05 and as trend at 0.05 < *p* < 0.10. All figures were made by GraphPad Prism 8. 

## 4. Conclusions

In the current study, according to the objective, the gene expression status of all nine known sodium/phosphate co-transporter genes was investigated in a broad range of pig tissues. In two independent pig trials, all P transporters exhibited a widely consistent expression pattern. Although the P transporters of the SLC17 family were not characterized as main actors in intestinal P absorption, *SLC17A2* and *SLC17A3* show a broad expression pattern in both peripheral and intestinal tissues of pigs. While SLC34A2 was considered as the most important intestinal P transporter in rodents and humans, *SLC34A3* showed a considerably higher abundance at the transcriptional level in the small intestine of pigs compared to *SLC34A2*. Therefore, the role of SLC34A2 in the intestines of pigs appears to be less pronounced compared to other species. However, further investigation of the protein expression level of the corresponding sodium/phosphate co-transporter might be of interest for further confirmation of these observations. Interestingly, seven and five out of nine sodium/phosphate transporters, including the important SLC34 family, were detectable in the distal colon of trial 1 and 2, respectively. Therefore, the distal colon might be also of relevance for intestinal inorganic P absorption. However, potential P transport functions of the distal colon still have to be confirmed experimentally. Regarding the responsiveness of P transporters to dietary P supply, two important P transporters, SLC34A1 and SLC34A3, showed higher gene expression in the low P group compared to the high P group in some kidney and intestinal tissues. Thus, it appears that the dietary regimen alters the level of expression of some P transporters in relevant tissues to maintain P homeostasis in the animal.

## Figures and Tables

**Table 1:** Gene information of sodium/phosphate co-transporters.

Gene	Gene Synonyms	Ensembl ID (v. 91)	Description
*SLC17A1*	*NAPI-1, NPT1*	ENSSSCG00000001107	Solute carrier family 17 member 1
*SLC17A2*	*NPT3*	ENSSSCG00000036191	Solute carrier family 17 member 2
*SLC17A3*	*NPT4*	ENSSSCG00000037547	Solute carrier family 17 member 3
*SLC17A4*	*NPT5*	ENSSSCG00000031944	Solute carrier family 17 member 4
*SLC34A1*	*NaPi-2a, NPT2a*	ENSSSCG00000037535	Solute carrier family 34 member 1
*SLC34A2*	*NaPi-2b, NPT2b*	ENSSSCG00000008758	Solute carrier family 34 member 2
*SLC34A3*	*NaPi-2c, NPT2c*	ENSSSCG00000040105	Solute carrier family 34 member 3
*SLC20A1*	*GLVR1, Glvr-1, PiT1*	ENSSSCG00000032288	Solute carrier family 20 member 1
*SLC20A2*	*GLVR2, Glvr-2, MLVAR, PiT2, Ram-1*	ENSSSCG00000007027	Solute carrier family 20 member 2

**Table 2:** Tissue samples and their specifications taken for both trials.

Tissue	Short	Specification	Trial ^1^
Kidney	Kidney cort	Cortex of left kidney	1, 2
Kidney	Kidney med	Medulla of left kidney	1, 2
Liver	Liver	Lobulus Spigelii	1
Stomach	Stomach	Fundus mucosa	1
Duodenum	Duod	Mucosa, 30–40 cm distal of pylorus	1, 2
Jejunum (prox.)	Jeju prox	Mucosa, 2 m distal of pylorus	1, 2
Jejunum (dist.)	Jeju dist	Mucosa, 2 m proximal of the ileocaecal junction	1, 2
Ileum	Ileum	Mucosa, 20 cm proximal of the ileocaecal junction	1, 2
Caecum	Caec	Mucosa	1, 2
Colon (prox.)	Colon prox	Mucosa, 50–60 cm distal of cecolic junction	1, 2
Colon (dist.)	Colon dist	Mucosa, 50–60 m proximal of rectum	1, 2
Bone	Bone	Calvarial bone, along the sagittal suture	1
Muscle	Muscle	Longissimus dorsi, between the 13th and 14th rib	1
Lung	Lung	Lower tip of the left lung lobe	1
Aorta	Aorta	Aorta, descending thoracic aorta	1

¹ Trial 1 represents pigs on a conventional standard diet and Trial 2 represents pigs on P divergent diets.

## Supplementary Materials

Supplementary materials can be found at https://www.mdpi.com/1422-0067/20/22/5576/s1.

## Abbreviations

P	Phosphorus
qRT-PCR	Quantitative real time polymerase chain reaction
SLC	Solute carrier
