# Supplementary material for: Tissue-Wide Gene Expression Analysis of Sodium/Phosphate Co-Transporters in Pigs
Source: Int J Mol Sci. 2019 Nov 8;20(22):5576. doi: 10.3390/ijms20225576 (PMC6888643; doi:10.3390/ijms20225576)
Supplement: Supplementary file 1 [file ijms-20-05576-s001.zip › Table S1.docx]

**Table S1. Sequence information of primers for qRT-PCR**

| **Primer** | **Sequence** | **Melting temperature (°C)** | **Amplicon length (bp)** | **Fragment type** |
| --- | --- | --- | --- | --- |
| RPL32f | AGCCCAAGATCGTCAAAAAG | 55.0 | 165 | qRT-PCR |
| RPL32r | TGTTGCTCCCATAACCAATG | 55.0 |  |  |
| RPL32f2 | TGCTCTCAGACCCCTTGTGA | 60.0 | 373 | Standard |
| RPL32r2 | ATTGGGATTGGTGACCCTGA | 60.0 |  |  |
| SSC_SLC17A1_E6_f | ATAAGCATCGGTGAGAAGGAATAC | 59.1 | 105 | qRT-PCR |
| SSC_SLC17A1_E7_r | GAGTGGAGGAGACTTAATCATAGCC | 60.1 |  |  |
| SSC_SLC17A1 _E3_f | GCTCTCTCTTTACCCTGCTCATC | 59.4 | 589 | Standard |
| SSC_SLC17A1 _E8_r | AAAATCCAGGCGAACAAATG | 57.8 |  |  |
| SSC_SLC17A2_E9_f | TTTTCACGGGGTTTTTCAGTC | 59.3 | 177 | qRT-PCR |
| SSC_SLC17A2_E10_r | GGAGGAAATCTGCCAACTGAC | 58.6 |  |  |
| SSC_SLC17A2 _E6_f | TACTGGCTGTGTCTGCTGTCTC | 57.8 | 599 | Standard |
| SSC_SLC17A2 _E10_r | GCCCAAATCCCCTTGAGATC | 60.7 |  |  |
| SSC_SLC17A3_E5_f | TGGGTGGTCTCATCAGTCAAG | 57.8 | 128 | qRT-PCR |
| SSC_SLC17A3_E6_r | TATCCAAGGGTGAGTGACAGG | 57.2 |  |  |
| SSC_SLC17A3 _E4_f | CAGGATATGGGGGTCAGTTTG | 59.3 | 369 | Standard |
| SSC_SLC17A3 _E7_r | GAAGGAAATCTGCCAGGTAGC | 58.3 |  |  |
| SSC_SLC17A4_E6_f | CCATCCTCTTTATTGGTGGTTTC | 59.7 | 123 | qRT-PCR |
| SSC_SLC17A4_E7_r | TGGGATCATCATAAACGAGAGC | 59.3 |  |  |
| SSC_SLC17A4 _E3_f | TATGGCTGAAATGTTTGGAGTC | 57.2 | 569 | Standard |
| SSC_SLC17A4 _E7_r | TGATGTATGTTGGTGTGTATGATG | 56.0 |  |  |
| SSC_SLC20A1_E6_f | CAGTTCCTACAGATCCTCACAGC | 58.3 | 174 | qRT-PCR |
| SSC_SLC20A1_E7_r | GCAGATACCAACACCACCATAG | 57.2 |  |  |
| SSC_SLC20A1 _E6_f | CAGAAAGGTGAAGAGATGGAGAAG | 59.3 | 585 | Standard |
| SSC_SLC20A1 _E9_r | GCCAACGGAGACAACAGAG | 55.8 |  |  |
| SSC_SLC20A2_E5_f | GTGGATGCGGAGGAAAATAG | 59.3 | 133 | qRT-PCR |
| SSC_SLC20A2_E6_r | ATTAGCCTTGGCACCTGGTAG | 56.6 |  |  |
| SSC_SLC20A2 _E3_f | GGCGTGCTGTTTGTACTGATC | 58.2 | 566 | Standard |
| SSC_SLC20A2 _E7_r | CCATTGGACACGGGTGAC | 56.6 |  |  |
| SSC_SLC34A1_E7_f | CTTGTGGTAGCCTCCTTCAAC | 56.1 | 125 | qRT-PCR |
| SSC_SLC34A1_E8_r | GTGGCAATGCTGGTAATCAC | 57.3 |  |  |
| SSC_SLC34A1 _E5_f | CTCCAACATCGGCACCTC | 56.2 | 554 | Standard |
| SSC_SLC34A1 _E9_r | AGTTGAGCATCTTGACGAGGAG | 58.7 |  |  |
| SSC_SLC34A2_E7_f | GAATCAGCCCGAAACAAGAG | 57.3 | 126 | qRT-PCR |
| SSC_SLC34A2_E8_r | ACCATCCGTCCAACAGAGG | 57.5 |  |  |
| SSC_SLC34A2 _E6_f | CGACTTCTTCAACTGGCTCTCC | 60.7 | 460 | Standard |
| SSC_SLC34A2 _E9_r | CAGGGCAGTTATCACCAGAATG | 59.7 |  |  |
| SSC_SLC34A3_E6_f | GCACGCACCAGACATCCTC | 59.1 | 135 | qRT-PCR |
| SSC_SLC34A3_E7_r | GTGCCGCACCATCTCTTAATG | 60.6 |  |  |
| SSC_SLC34A3 _E5_f | GTGTCAACGTGGGCACATC | 56.9 | 572 | Standard |
| SSC_SLC34A3 _E9_r | GTTGAGCAGCTTGACGATGAG | 58.3 |  |  |
|  |  |  |  |  |
