# Supplementary material for: Tissue-Wide Gene Expression Analysis of Sodium/Phosphate Co-Transporters in Pigs
Source: Int J Mol Sci. 2019 Nov 8;20(22):5576. doi: 10.3390/ijms20225576 (PMC6888643; doi:10.3390/ijms20225576)
Supplement: Supplementary file 1 [file ijms-20-05576-s001.zip › Table S2.docx]

Table S2. Transcript copy numbers of differentially expressed Sodium/phosphate cotransporters in pigs receiving divergent P-containing diets (Trial 2).

| **Gene** | **Tissue^1^** | **FC^2^** | **p-value** | **Transcript copy number** | | |
| --- | --- | --- | --- | --- | --- | --- |
|  |  |  |  | Mean | Min | Max |
| SLC17A1 | kidney cortex | -1.42 | 0.53 | 25772.06 | 8629.60 | 61224.53 |
|  | kidney med | 1.03 | 0.83 | 2299.36 | 251.58 | 17397.24 |
| SLC17A2 | caecum | -1.34 | 0.24 | 14.68 | 6.65 | 28.94 |
|  | jejunum distal | -2.073 | 0.25 | 10.28 | 2.52 | 43.48 |
|  | duodenum | 1.43 | 0.50 | 5.17 | 1.95 | 12.27 |
|  | kidney cortex | -1.49 | 0.68 | 221.37 | 26.71 | 675.22 |
|  | ileum | 1.19 | 0.72 | 15.08 | 4.28 | 30.40 |
|  | colon distal | 1.06 | 0.79 | 6.73 | 1.80 | 13.68 |
|  | jejunum proximal | 1.04 | 0.83 | 5.65 | 1.62 | 9.16 |
|  | kidney med | 1.24 | 0.95 | 50.375 | 8.82 | 265.95 |
| **SLC17A3** | **kidney cortex** | **-5.58** | ***0.08*** | ***69858.38*** | ***316.02*** | ***197199.2*** |
|  | caecum | -1.81 | 0.20 | 49.58 | 6.52 | 163.02 |
|  | kidney med | -1.95 | 0.36 | 6100.72 | 355.36 | 40355.89 |
|  | ileum | 2.09 | 0.43 | 42.57 | 2.37 | 141.76 |
|  | colon distal | -1.51 | 0.48 | 27.23 | 4.83 | 70.81 |
|  | jejunum proximal | -1.36 | 0.53 | 6.49 | 0.84 | 12.87 |
|  | duodenum | 1.58 | 0.75 | 30.40 | 2.31 | 78.66 |
|  | jejunum distal | -1.26 | 0.81 | 32.47 | 1.64 | 157.18 |
|  | colon proximal | 1.03 | 0.85 | 6.89 | 1.65 | 29.16 |
| SLC17A4 | kidney cortex | 2.24 | 0.17 | 131.99 | 16.74 | 375.41 |
|  | kidney med | -1.20 | 0.57 | 19.69 | 1.78 | 100.66 |
| **SLC20A1** | **kidney cortex** | **-3.19** | ***0.00*** | ***2314.96*** | ***639.97*** | ***5177.85*** |
|  | **caecum** | **-1.61** | ***0.02*** | ***8326.76*** | ***3652.06*** | ***17206.72*** |
|  | colon proximal | -1.73 | 0.14 | 944.18 | 225.09 | 1896.21 |
|  | ileum | 2.24 | 0.19 | 5007.28 | 590.69 | 10385.58 |
|  | jejunum distal | 1.57 | 0.42 | 6163.85 | 1433.67 | 15419.63 |
|  | duodenum | -1.10 | 0.55 | 1195.63 | 461.41 | 2192.86 |
|  | jejunum proximal | -1.42 | 0.60 | 1503.96 | 465.99 | 5948.92 |
|  | kidney med | -1.08 | 0.80 | 2433.95 | 508.93 | 5524.16 |
|  | colon distal | 1.07 | 0.88 | 3448.10 | 1014.42 | 7188.42 |
| **SLC20A2** | **jejunum distal** | **2.89** | ***0.01*** | ***1259.51*** | ***264.37*** | ***3308.09*** |
|  | **kidney cortex** | **-3.09** | ***0.04*** | ***450.46*** | ***74.51*** | ***1366.18*** |
|  | **colon proximal** | **-1.76** | ***0.05*** | ***426.06*** | ***127.91*** | ***716.62*** |
|  | **ileum** | **2.31** | ***0.08*** | ***706.68*** | ***154.47*** | ***2011.38*** |
|  | caecum | -1.38 | 0.26 | 852.23 | 362.59 | 1862.46 |
|  | kidney med | 1.30 | 0.31 | 485.51 | 299.80 | 819.95 |
|  | jejunum proximal | -1.40 | 0.54 | 322.40 | 146.11 | 1268.73 |
|  | colon distal | -1.00 | 0.58 | 567.61 | 298.09 | 780.25 |
|  | duodenum | 1.21 | 0.65 | 208.64 | 115.53 | 445.21 |
| **SLC34A1** | **kidney cortex** | **-2.93** | ***0.01*** | ***434571.8*** | ***140222.6*** | ***1117785*** |
|  | kidney med | -1.05 | 0.78 | 18319.34 | 1864.27 | 104953.8 |
| SLC34A2 | ileum | 5.38 | 0.22 | 5.45 | 0.02 | 13.46 |
|  | kidney med | -1.99 | 0.30 | 121.64 | 24.45 | 539.40 |
|  | kidney cortex | -1.65 | 0.32 | 22.21 | 4.73 | 55.80 |
|  | colon distal | 1.35 | 0.38 | 4.63 | 2.07 | 7.55 |
|  | jejunum distal | 2.81 | 0.42 | 4.37 | 0.04 | 16.07 |
|  | caecum | 1.13 | 0.61 | 5.68 | 2.47 | 15.84 |
| **SLC34A3** | **ileum** | **-7.78** | ***0.00*** | ***927.80*** | ***12.55*** | ***5812.77*** |
|  | **kidney cortex** | **-2.69** | ***0.01*** | ***2769.50*** | ***714.86*** | ***6116.81*** |
|  | **jejunum distal** | **-2.63** | ***0.08*** | ***5613.20*** | ***1458.84*** | ***24500.05*** |
|  | duodenum | -1.89 | 0.15 | 1302.75 | 495.99 | 4304.56 |
|  | jejunum proximal | -1.70 | 0.37 | 5919.12 | 1137.64 | 17997.15 |
|  | kidney med | -1.14 | 0.71 | 192.49 | 37.45 | 670.22 |

¹Gene expression in tissues which are not presented was not detectable.

^2^ Fold change in gene expression between low and high P diet groups.
